# Supplementary material for: Identification and Preliminary Analysis of Granulosa Cell Biomarkers to Predict Oocyte In Vitro Maturation Outcome in the Southern White Rhinoceros (Ceratotherium simum simum)
Source: Animals (Basel). 2024 Dec 7;14(23):3538. doi: 10.3390/ani14233538 (PMC11640850; doi:10.3390/ani14233538)
Supplement: Supplementary file 1 [file animals-14-03538-s001.zip › animals-3337820-supplementary.pdf]

	PB					
	PB1	PB2	PB3	PB4	PB5	PB6
COL1A1	-2.267	-2.724	0.706	-2.618	-2.352	-2.463
GDF9	0.364	0.535	0.706	0.042	0.15	0.258
KAT8	0.526	0.775	0.121	0.61	0.889	0.585
LHR	3.383	4.298	4.467	4.022	4.703	4.798
MTOR	-5.471	-5.457	-5.443	-5.553	-5.544	-5.534
PGR	0.092	0.606	0.259	0.967	0.721	0.02
TNF	3.236	0.567	3.733	2.928	0.964	3.466
TP53	2.625	4.713	4.068	3.701	4.439	5.766
FBXW11	0.909	0.216	0.635	0.485	0.684	0.565
GGPS1	-5.495	-5.509	-5.694	-5.665	-5.619	-5.915
JMY	0.359	0.475	0.591	0.136	0.079	0.482
MVK	0.962	1.595	0.521	0.738	1.355	0.816
NPR2	-0.009	0.051	0.11	0.07	0.181	0.292
NRG1	0.259	0.414	-0.166	0.09	0.649	-0.448
COL4A1	0.696	1.007	0.063	0.218	0.223	0.179
MACIR	3.247	3.415	3.583	3.699	3.938	4.178
TMPO	1.042	0.969	0.895	0.926	1.012	1.098
BCL2A1	1.335	0.569	0.713	1.044	0.453	1.03
CCT3	-0.758	-0.517	-0.964	-0.805	-0.105	-0.682
HNRNPA2B	0.657	0.365	0.293	0.195	1.121	0.075
MYC	-0.064	-0.638	-0.428	-0.498	-0.879	-0.085
NFYA	1.199	4.082	1.261	4.794	4.89	4.986

no PB

noPB1	noPB2	noPB3	noPB4	noPB5	noPB6
-1.989	-1.93	-2.155	-1.974	-2.073	-2.172
2.085	2.534	2.956	4.677	3.085	3.047
0.591	0.147	0.82	0.011	0.645	0.652
4.316	4.409	4.124	3.051	2.689	4.258
1.129	1.16	1.057	0.387	0.864	0.748
0.492	-0.027	0.363	-0.484	0.509	0.471
0.45	2.752	3.251	2.893	3.654	0.805
4.638	5.015	4.157	3.531	3.98	4.145
0.366	0.534	0.902	0.027	0.878	0.283
1.171	1.045	1.297	1.371	1.357	1.364
1.283	0.81	0.337	0.743	0.687	0.92
1.365	0.489	1.43	0.089	0.998	1.514
1.364	1.168	1.385	1.044	1.04	1.036
0.432	-0.177	0.015	-0.187	0.015	0.66
-7.23	-7.168	-7.292	-7.632	-7.415	-7.524
0.335	0.502	0.419	0.245	0.329	0.312
-7.079	-7.136	-7.669	-7.761	-7.397	-7.641
0.485	1.183	0.574	0.624	0.79	0.584
-0.434	-1.062	-0.621	-0.96	-1.025	-0.248
0.459	0.241	0.376	-0.267	0.34	0.657
-0.678	-0.223	-0.008	-0.825	-0.128	-1.036
0.316	0.443	-0.177	0.099	0.607	-0.286
